# Facial Lifting and Skin Rejuvenation Using a Dual‐Wavelength Picosecond Nd:YAG Laser in Asian Skin: A Prospective Pilot Study

**DOI:** 10.1111/jocd.71206

**Published:** 2026-09-20

**Authors:** Yoon Hwan Lee, Sin Woo Sung, Ji Yeon Hong, Kui Young Park

**Affiliations:** ^1^ Department of Dermatology Chung‐Ang University College of Medicine Seoul Republic of Korea

**Keywords:** Asian skin, dermal remodeling, dual‐wavelength picosecond laser, Nd:YAG laser, skin laxity, skin rejuvenation

## Abstract

**Background:**

Fractional picosecond lasers promote nonablative skin rejuvenation by inducing dermal remodeling through photomechanical effects. However, prospective clinical evidence regarding dual‐wavelength picosecond Nd:YAG laser systems remains limited.

**Objective:**

To evaluate the efficacy and safety of a dual‐wavelength picosecond Nd:YAG laser for facial laxity and skin quality.

**Methods:**

In this prospective, single‐arm pilot study, 20 adults underwent three treatments at 4‐week intervals using sequential 1064‐ and 532‐nm picosecond laser. Outcomes included Cutometer parameters (R2, R5, R7), melanin index (MI), erythema index (EI), imaging analysis, physician‐assessed GAIS, participant satisfaction, and adverse events.

**Results:**

All participants completed the study. R5 and R7 improved from Week 4 onward, while R2 improved significantly at Weeks 8 and 12. MI and EI decreased significantly at Week 12. Imaging analysis showed significant improvements in pores, wrinkles, pigmentation, skin tone, and gloss. Week‐12 GAIS and participant satisfaction scores were 4.70 ± 0.57 and 4.50 ± 0.76, respectively. Adverse events were limited to transient erythema and transient worsening of preexisting malar melasma in two participants, which resolved spontaneously.

**Conclusion:**

Dual‐wavelength picosecond Nd:YAG laser treatment progressively improved skin elasticity, skin tone, and overall skin quality with a favorable safety profile, supporting its potential for nonablative facial lifting and skin rejuvenation.

## 1 Introduction

Facial skin laxity is one of the most prominent manifestations of skin aging and represents a major concern among individuals seeking aesthetic treatment. Age‐related degradation of dermal collagen and elastic fibers, together with alterations in the extracellular matrix (ECM), leads to reduced skin elasticity, impaired biomechanical properties, enlarged pores, fine wrinkles, uneven skin texture, and loss of skin radiance [1, 2, 3]. Because these structural changes primarily involve the dermis, treatment strategies that promote dermal remodeling have become a major focus in aesthetic dermatology.

Picosecond‐domain lasers were initially developed for the treatment of tattoos and pigmented lesions because of their ultrashort pulse duration and extremely high peak power. Compared with conventional nanosecond lasers, picosecond lasers predominantly generate photomechanical rather than photothermal effects, producing highly localized tissue disruption with minimal collateral thermal injury [4, 5, 6]. These characteristics have expanded their clinical application beyond pigment clearance to nonablative skin rejuvenation through stimulation of dermal remodeling.

The introduction of diffractive optical element (DOE)‐based fractional beam delivery has further broadened the applications of picosecond lasers. Fractional picosecond irradiation generates laser‐induced optical breakdown (LIOB), characterized by microscopic intradermal cavitation with minimal surrounding thermal damage. Experimental and histologic studies have demonstrated that LIOB initiates wound‐healing responses involving fibroblast activation, neocollagenesis, neoelastogenesis, and ECM remodeling, ultimately leading to improvements in skin elasticity, wrinkle appearance, pore size, and overall skin quality [7, 8, 9, 10]. Recent in vivo confocal and histopathologic studies have further confirmed controlled dermal LIOB formation and subsequent tissue remodeling following fractional picosecond laser treatment [9, 10].

Clinical studies have demonstrated favorable outcomes of fractional picosecond lasers for improving facial photoaging, including skin laxity, enlarged pores, wrinkles, acne scars, dyspigmentation, and overall skin texture, particularly in Asian skin. However, treatment outcomes remain heterogeneous because of differences in wavelength, pulse duration, beam delivery systems, and treatment protocols. Moreover, prospective clinical evidence regarding dual‐wavelength picosecond Nd:YAG laser systems capable of simultaneously targeting dermal remodeling and superficial skin quality remains limited.

SCULPIO (Laseroptek Co. Ltd., Seongnam, Republic of Korea) is a dual‐wavelength (1064/532 nm) picosecond Nd:YAG laser platform incorporating proprietary PicoSculpting technology with DOE‐based fractional beam delivery. The 1064‐nm wavelength is designed to induce depth‐adjustable dermal LIOB for collagen and elastic fiber remodeling, whereas the fractional 532‐nm wavelength targets superficial skin quality while minimizing epidermal injury. This complementary dual‐wavelength approach may provide broader improvement in skin laxity and overall skin quality within a single treatment session.

Therefore, the present prospective pilot study aimed to evaluate the efficacy and safety of a dual‐wavelength picosecond Nd:YAG laser for the treatment of facial skin laxity and skin rejuvenation using objective biophysical measurements, imaging‐based skin analysis, and physician‐ and participant‐reported clinical outcomes.

## 2 Materials and Methods

### 2.1 Study Design and Setting

This was a single‐center, single‐arm, prospective, open‐label pilot study conducted at the Department of Dermatology, Chung‐Ang University Hospital, Seoul, Republic of Korea. Twenty participants with clinically apparent facial photoaging were prospectively enrolled and underwent three treatment sessions at 4‐week intervals, followed by a 4‐week posttreatment follow‐up. The study protocol was approved by the Institutional Review Board of Chung‐Ang University Hospital (IRB No. 2506‐016‐647) and was conducted in accordance with the ethical principles of the Declaration of Helsinki [11]. Written informed consent was obtained from all participants before study enrollment, including consent for clinical photography and publication of de‐identified images.

### 2.2 Participants

Adults aged 20–80 years with facial skin laxity and clinically apparent signs of skin aging, including decreased skin elasticity, enlarged pores, fine wrinkles, and deterioration of skin texture, were eligible for enrollment. Participants were required to be in good general health and willing to comply with the study protocol and follow‐up schedule.

Key exclusion criteria included pregnancy or lactation, a history of keloid or hypertrophic scar formation, active inflammatory or infectious facial dermatoses, photosensitivity disorders, severe uncontrolled systemic disease, receipt of facial aesthetic procedures during the study period, or any medical condition that, in the investigator's opinion, could interfere with study participation or outcome assessment.

### 2.3 Laser Device and Treatment Protocol

The investigational device was SCULPIO (Laseroptek Co. Ltd., Seongnam, Republic of Korea), a dual‐wavelength picosecond Nd:YAG laser platform equipped with 1064 and 532‐nm wavelengths. The system incorporates proprietary PicoSculpting (PS) technology combined with a DOE‐based fractional beam delivery system. The DOE divides the incident laser beam into a fractional microbeam array, enabling controlled LIOB within the dermis while minimizing collateral thermal injury. The 1064‐nm PS handpiece provides adjustable LIOB formation depths, with Depth Level I targeting the lower dermis and Depth Level II targeting the upper dermis, whereas the 532‐nm PS handpiece is designed to induce intradermal fractional LIOB using low‐fluence fractional energy delivery while minimizing epidermal injury. The pulse durations were 450 ps at 1064 nm and 380 ps at 532 nm.

Each participant underwent three full‐face treatment sessions at 4‐week intervals using two sequential treatment modes. First, the 1064‐nm PS mode was delivered using an 11 × 11 mm^2^ PS handpiece at Depth Level I in S2 stamping mode (20 repetitive shots over 2 s) at 0.23 J/cm^2^ to induce dermal remodeling. This was followed by 532‐nm PS treatment using an 8 × 8 mm^2^ PS handpiece with a painting (linear) technique at 10 Hz and 0.04 J/cm^2^ to improve superficial skin quality. Clinical evaluations were performed at baseline and at Weeks 4, 8, and 12. No concurrent facial energy‐based procedures or other aesthetic treatments were permitted during the study period.

### 2.4 Outcome Measures

Objective efficacy outcomes included Cutometer‐derived elasticity parameters and Mexameter‐derived skin tone parameters. Cutometer measurements (Cutometer MPA 580, Courage + Khazaka, Cologne, Germany) included R2 (gross elasticity, Ua/Uf), R5 (net elasticity, Ur/Ue), and R7 (elastic recovery ratio, Ur/Uf), with values expressed as percentages (ratio×100).

Mexameter measurements (Mexameter MX 18, Courage + Khazaka) included melanin index (MI) and erythema index (EI). Cutometer and Mexameter measurements were performed under standardized environmental conditions (temperature 20°C–22°C, relative humidity 40%–60%), following a 20‐min acclimatization period. All measurements were performed at predefined facial sites on both cheeks, with three consecutive measurements obtained on each side. The mean of the six measurements was used for analysis.

Standardized facial images were acquired using a Mark‐Vu skin analysis system (PSI Plus Co. Ltd., Republic of Korea) under consistent lighting and positioning conditions for two‐dimensional assessment of skin parameters, including brown pigmentation, pores, wrinkles, pigmentation, sebum, skin tone, and gloss. Assessments were performed at baseline and at each follow‐up visit.

Subjective outcomes included physician‐assessed Global Aesthetic Improvement Scale (GAIS) and participant satisfaction scores. GAIS was assessed using a 5‐point scale ranging from 1 (worse) to 5 (very much improved), and participant satisfaction was evaluated using a 5‐point scale ranging from 1 (very dissatisfied) to 5 (very satisfied). Given the consistently high scores and potential ceiling effects, these subjective measures were interpreted as supportive descriptive endpoints.

### 2.5 Safety Evaluation

Safety was assessed by direct investigator evaluation of treatment‐related adverse events, including erythema, edema, petechiae, pigmentary changes, and other local reactions, as well as any serious adverse events. Safety was monitored at each visit throughout the study.

### 2.6 Statistical Analysis

Normality of the paired differences between baseline and each follow‐up time point was assessed using the Shapiro–Wilk test. As the paired differences satisfied the normality assumption, baseline‐to‐follow‐up comparisons were performed using paired *t*‐tests. Data are presented as mean ± standard deviation. Given the exploratory nature of this pilot study, *p* values were not adjusted for multiple comparisons and should be interpreted descriptively. All statistical tests were two‐sided, and *p* values < 0.05 were considered statistically significant.

## 3 Results

### 3.1 Participant Characteristics

Twenty participants were enrolled, and all completed the study and were included in the final analysis. The mean age was 57.55 ± 9.07 years. Nineteen participants (95.0%) were female, and one (5.0%) was male. Fitzpatrick skin phototypes were III in 11 participants (55.0%) and IV in 9 participants (45.0%). Preexisting malar melasma was present in 8 participants (40.0%).

### 3.2 Changes in Skin Elasticity

Cutometer measurements demonstrated a time‐dependent improvement in skin elasticity parameters (Table 1). The overall elasticity parameter (R2) increased from 46.4 ± 12.5 at baseline to 47.6 ± 11.6 at Week 4, 49.6 ± 10.0 at Week 8, and 52.7 ± 9.0 at Week 12. Compared with baseline, the increase was not statistically significant at Week 4 (*p* = 0.337), but became significant at Week 8 (*p* = 0.041) and more pronounced at Week 12 (*p* < 0.001). The net elasticity parameter (R5) showed a significant increase at all follow‐up time points, rising from 30.8 ± 11.5 at baseline to 34.9 ± 12.4 at Week 4, 35.9 ± 10.1 at Week 8, and 39.9 ± 9.4 at Week 12. The corresponding *p* values versus baseline were 0.003, 0.003, and < 0.001, respectively. Similarly, the biological elasticity parameter (R7) increased from 23.3 ± 8.7 at baseline to 28.3 ± 7.7 at Week 4, 32.5 ± 9.3 at Week 8, and 33.5 ± 8.8 at Week 12, with statistically significant improvements at all follow‐up time points (all *p* < 0.001). Collectively, these findings suggest early and sustained gains in elastic recoil with a delayed but significant increase in overall elasticity.

**TABLE 1:** Changes in Cutometer parameters over time.

Parameter	Baseline	Week 4	Week 8	Week 12
R2	46.4 ± 12.5	47.6 ± 11.6	49.6 ± 10.0*	52.7 ± 9.0***
R5	30.8 ± 11.5	34.9 ± 12.4**	35.9 ± 10.1**	39.9 ± 9.4***
R7	23.3 ± 8.7	28.3 ± 7.7***	32.5 ± 9.3***	33.5 ± 8.8***

*Note:* Data are presented as mean ± standard deviation. Statistical significance was assessed using paired *t*‐tests compared with baseline.

* *p* < 0.05.

** *p* < 0.01.

*** *p* < 0.001.

### 3.3 Changes in Skin Tone Parameters

**FIGURE 1:**
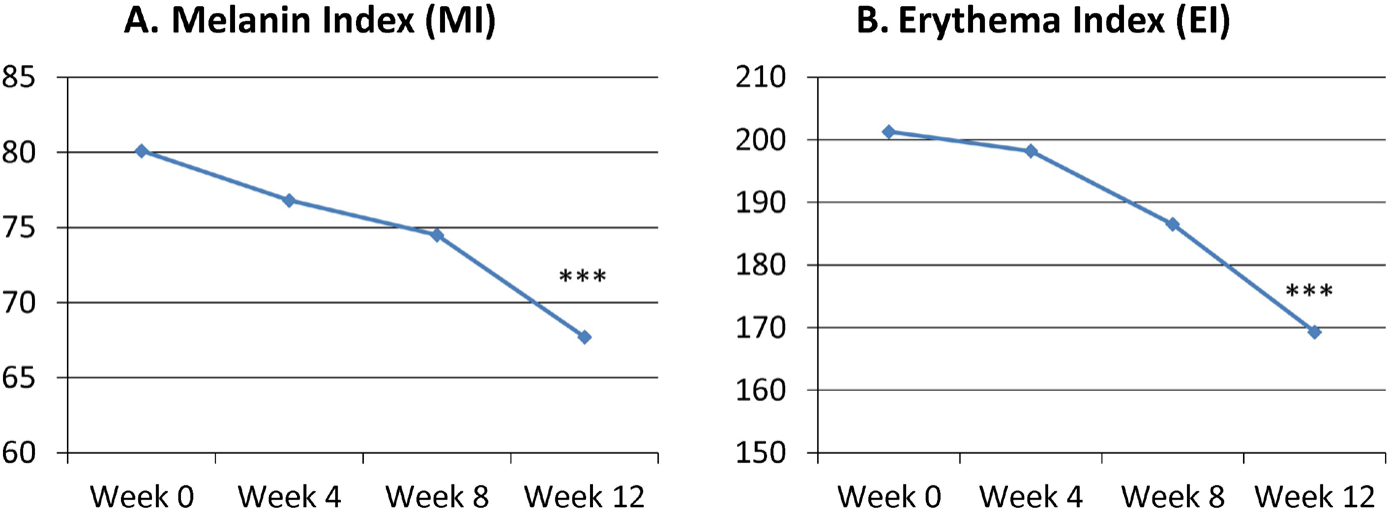
Changes in melanin index and erythema index over time. (A) Melanin index (MI) and (B) erythema index (EI) measured using Mexameter at baseline and at Weeks 4, 8, and 12. Both indices showed a gradual decrease over time, with statistically significant reductions observed at Week 12 compared with baseline (***p < 0.001). Data are presented as mean ± standard deviation.

Mexameter measurements demonstrated progressive improvement in skin tone parameters over time. MI decreased from 80.1 ± 26.9 at baseline to 76.8 ± 29.1 at Week 4, 74.5 ± 32.1 at Week 8, and 67.7 ± 27.1 at Week 12. The reduction was not statistically significant at Week 4 (*p* = 0.193) or Week 8 (*p* = 0.087), but reached significance at Week 12 (*p* < 0.001) (Figure 1A). EI showed a similar delayed pattern, decreasing from 201.3 ± 53.7 at baseline to 198.2 ± 63.2 at Week 4, 186.5 ± 62.8 at Week 8, and 169.3 ± 55.6 at Week 12. The Week‐12 decrease versus baseline was statistically significant (*p* < 0.001), whereas changes at Weeks 4 and 8 were not (Figure 1B).

### 3.4 Imaging‐Based Skin Analysis

**FIGURE 2:**
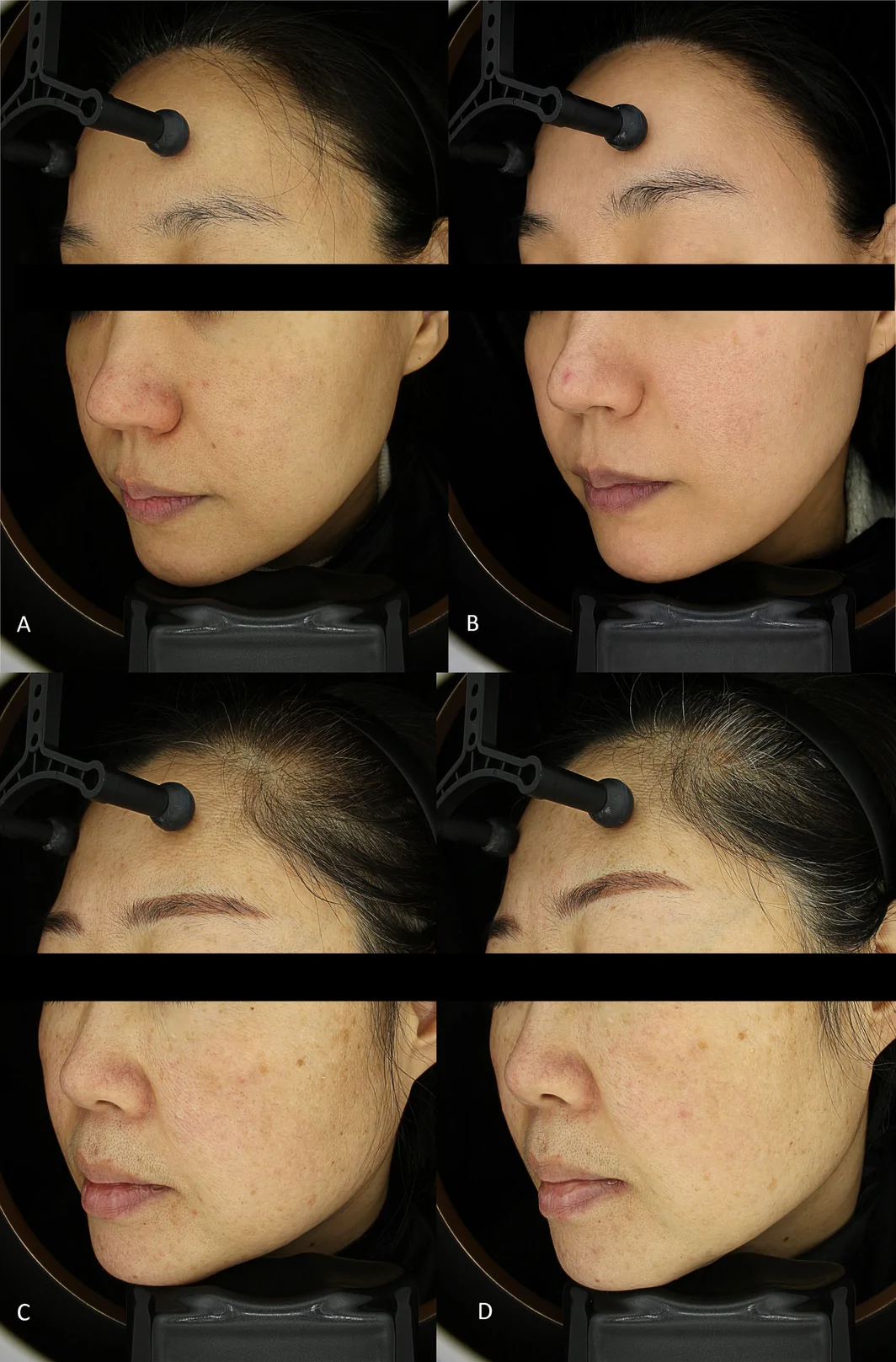
Representative clinical photographs showing changes potentially consistent with facial lifting and skin rejuvenation following treatment with the dual‐wavelength picosecond Nd:YAG laser. Representative clinical photographs obtained at baseline (A, C) and 4 weeks after the final treatment (B, D). In the first participant (A, B), visual improvement in the nasolabial fold, facial contour, and overall skin tone was observed. In the second participant (C, D), visual improvement in jawline definition and overall skin tone was evident. All participants underwent three treatment sessions at 4‐week intervals.

Imaging‐based skin analysis demonstrated significant changes in several skin parameters over the treatment period. Pore_Avg decreased significantly from baseline at Visits 2, 3, and 4 (all *p* < 0.05). Wrinkle_Avg showed a gradual decrease and was significantly reduced at Visits 3 and 4 (both *p* < 0.05). Pigmentation_Avg also decreased significantly at Visits 3 and 4 compared with baseline (both *p* < 0.05). SkinTone_Avg showed significant increases at Visits 3 and 4 (both *p* < 0.05). Similarly, Gloss_Avg increased significantly at Visits 3 and 4 (both *p* < 0.01) (Table 2). In contrast, Brown_Avg and Sebum_Avg did not show statistically significant changes during the study period (all *p* > 0.05). Representative clinical photographs are shown in Figure 2.

**TABLE 2:** Imaging‐based skin analysis during the study period.

Parameter	Baseline	Visit 2	*p* *	Visit 3	*p* *	Visit 4	*p* *
Brown pigmentation score	23.60 ± 5.39	23.80 ± 5.53	0.592	23.75 ± 5.38	0.754	23.75 ± 5.60	0.720
Pore score	58.90 ± 5.45	56.95 ± 5.87	0.007	55.35 ± 5.44	0.003	53.45 ± 6.08	< 0.001
Wrinkle score	28.95 ± 2.87	28.05 ± 3.02	0.104	27.40 ± 2.80	0.044	26.05 ± 3.66	< 0.001
Pigmentation score	37.20 ± 7.88	36.80 ± 7.02	0.596	35.00 ± 7.85	0.036	32.75 ± 8.54	< 0.001
Sebum score	17.85 ± 12.47	17.85 ± 12.15	1.000	17.30 ± 11.76	0.828	18.40 ± 12.34	0.719
Skin tone score	55.00 ± 3.34	55.35 ± 3.03	0.110	56.00 ± 3.18	0.014	57.35 ± 4.07	0.002
Gloss score	20.35 ± 14.27	21.40 ± 13.32	0.085	23.70 ± 15.00	< 0.001	25.95 ± 17.40	< 0.001

*Note:* Values are presented as mean ± standard deviation.

* Compared with baseline using paired *t*‐test.

### 3.5 Subjective Outcomes

**FIGURE 3:**
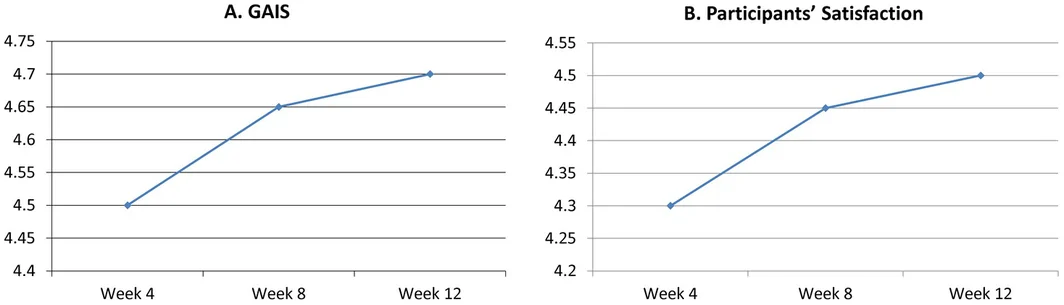
Physician‐assessed GAIS and patient‐reported satisfaction over time. (A) Physician‐assessed Global Aesthetic Improvement Scale (GAIS) scores and (B) patient‐reported satisfaction scores at Weeks 4, 8, and 12. Both outcomes demonstrated consistently high values from the earliest posttreatment time point, with slight increasing trends over time. Data are presented as mean ± standard deviation.

Subjective assessments demonstrated consistently high levels of perceived improvement and satisfaction throughout follow‐up. Physician‐assessed GAIS increased from 4.50 ± 0.69 at Week 4 to 4.65 ± 0.59 at Week 8 and 4.70 ± 0.57 at Week 12. Participant‐reported satisfaction scores were 4.30 ± 0.73 at Week 4, 4.45 ± 0.76 at Week 8, and 4.50 ± 0.76 at Week 12 (Figure 3). Because both scales exhibited high initial values and a limited ordinal range, these outcomes were interpreted descriptively rather than as primary inferential endpoints.

### 3.6 Safety

Treatment‐related adverse events were limited to transient erythema, which resolved spontaneously without sequelae. Two participants experienced transient worsening of preexisting malar melasma, which improved spontaneously during follow‐up without additional treatment. No serious adverse events were observed.

## 4 Discussion

This prospective pilot study suggests that treatment with the dual‐wavelength SCULPIO picosecond Nd:YAG laser may improve multiple dimensions of facial skin quality over a 12‐week course. Objective gains were most robust for Cutometer‐derived elastic recoil parameters (R5 and R7), while R2 improved later, and Mexameter‐derived tone parameters (MI and EI) showed delayed but significant improvement by Week 12.

The elasticity data demonstrated a distinct temporal pattern. R5 and R7, which reflect net elasticity and elastic recovery, improved from the first post‐baseline assessment onward. In contrast, R2, a broader indicator of gross elasticity, became significant only after repeated treatment. These findings indicate that different aspects of skin elasticity may respond differently over the treatment course, although the biological basis for these temporal differences remains unclear.

Clinically, these changes may be consistent with a potential nonablative lifting effect rather than simple surface rejuvenation, because improved recoil and gross elasticity are biomechanically relevant to facial firmness and contour.

A likely mechanistic explanation involves the high‐peak‐power photomechanical effect of picosecond pulses, particularly when coupled with fractional beam delivery optics. Fractional picosecond irradiation can generate LIOB and laser‐induced cavitation (LIC) within epidermal and dermal targets, producing microscopic zones of tissue disruption with limited surrounding thermal injury [12]. Ex vivo and in vivo histologic studies have demonstrated vacuolar changes and dermal microinjury patterns after fractional picosecond irradiation, while reflectance confocal microscopy and histopathologic correlation studies have further confirmed controlled LIOB formation in human skin [7, 8, 9]. These controlled microinjuries are thought to initiate wound‐healing responses that lead to fibroblast activation, neocollagenesis, neoelastogenesis, and extracellular matrix remodeling [12, 13]. An animal study using a fractional 1064‐nm picosecond laser demonstrated increased dermal thickness and upregulation of collagen synthesis markers after treatment, providing molecular biologic support for the dermal remodeling mechanism underlying clinical rejuvenation [13].

The observed improvement in elasticity is also consistent with prior clinical studies of fractional picosecond lasers. Fractional 1064‐nm picosecond Nd:YAG laser treatment using a DOE has been reported to improve facial wrinkles and acne scars with favorable safety [14]. More recently, fractional 1064‐nm Nd:YAG picosecond laser treatment in Asian patients was reported to improve indices of skin rejuvenation and was well tolerated, supporting its applicability in darker skin phototypes [15]. In vivo evaluation with reflectance confocal microscopy has also shown clinical and microscopic improvement in photoaging after fractional 1064‐nm picosecond laser treatment [16]. Collectively, these studies support the concept that fractional picosecond‐domain treatment can improve skin aging through dermal remodeling rather than pigment clearance alone.

An important feature of the present protocol is the complementary use of 1064‐nm and 532‐nm wavelengths within a single treatment session. The 1064‐nm PS mode was intended primarily to induce dermal remodeling and improve biomechanical properties, whereas the 532‐nm PS mode was used to target superficial skin quality, including pigmentation and skin tone. A randomized controlled split‐face study comparing 1064‐nm and dual‐wavelength 532/1064‐nm picosecond‐domain Nd:YAG lasers demonstrated that both approaches improved photodamaged facial skin [17]. In addition, a randomized prospective split‐face pilot study evaluating 532 and 1064‐nm picosecond Nd:YAG lasers using DOE fractional technology reported improvement in photoaged skin, with 532‐nm treatment showing particular benefit for epidermal pigmentary lesions [18]. Although the present study was not designed to determine the independent contribution of each wavelength, the combination of early elasticity improvement, delayed MI/EI improvement, and favorable imaging‐based changes is compatible with this complementary treatment strategy.

The delayed improvements in MI and EI may reflect the different temporal biology of tone normalization compared with elastic recovery. Picosecond‐domain lasers exert selective photomechanical effects on pigment‐containing structures while minimizing nonspecific thermal damage, which is particularly relevant in Asian skin [4, 5, 15]. Fractional treatment may also alter dermal optical properties through remodeling of collagen and ECM architecture, which could contribute to gradual improvement in skin tone, erythema index, and visible brightness. The Week‐12 significance for both MI and EI therefore appears coherent with the cumulative three‐session protocol used in this study, rather than indicating an immediate pigment‐clearing or vascular effect after a single session. The imaging‐based analysis further supports the multidimensional nature of the treatment response. Pore score decreased significantly from the early follow‐up visits, whereas wrinkle and pigmentation scores showed more gradual improvement. Skin tone and gloss scores also increased significantly at later visits. These findings align with previous reports showing that fractional picosecond lasers can improve photoaging‐associated concerns such as pores, wrinkles, texture irregularity, dyspigmentation, and overall skin quality [12, 14, 15, 18]. In the present study, the parallel improvement in elasticity, tone parameters, and imaging‐based skin‐quality indices suggests that the treatment effect was not limited to a single domain of skin aging.

The high GAIS and participant‐satisfaction scores complement the objective findings but should be interpreted cautiously. Because these scales were already high from the earliest follow‐up, a ceiling effect is likely, and their ordinal structure limits fine‐grained discrimination across visits. Nonetheless, the consistently favorable physician‐ and patient‐reported outcomes support the clinical relevance of the observed objective improvements.

Our findings align with prior reports showing that fractional picosecond lasers can improve photoaging‐associated concerns such as pores, wrinkles, texture irregularity, and dyschromia, particularly in Asian skin. However, direct comparison across devices remains difficult because prior studies differ in wavelength, pulse structure, optics, treatment density, delivery pattern, and endpoint selection. The present study adds device‐specific prospective data for a dual‐wavelength 532/1064‐nm picosecond Nd:YAG platform using a combined full‐face protocol.

Overall, treatment was well tolerated, and treatment‐related adverse events were limited to transient erythema that resolved spontaneously without sequelae. However, transient worsening of preexisting malar melasma was observed in two participants, both of whom improved spontaneously during follow‐up without additional treatment. Although a causal relationship cannot be established in this pilot study, these observations highlight the need for caution when treating patients with preexisting melasma, particularly in the malar region. In clinical practice, reducing the treatment fluence, minimizing pulse overlap, or avoiding direct irradiation of active melasma lesions may help reduce the risk of transient exacerbation. Further studies are warranted to establish optimal treatment parameters for patients with concomitant melasma.

This study has several limitations. First, it was a small single‐arm pilot study performed at a single center without a comparator or sham control. Second, follow‐up was limited to 4 weeks after the final treatment, preventing assessment of longer‐term durability. Third, although objective instrumentation and imaging‐based analysis strengthened the dataset, the lifting effect was inferred from Cutometer‐derived elasticity parameters and representative clinical photographs rather than direct three‐dimensional volumetric or contour analysis. Fourth, histologic confirmation was not performed in this cohort; therefore, the proposed dermal remodeling mechanism should be interpreted as biologically plausible but not directly proven in the study participants. Fifth, photoprotective behaviors and topical skincare regimens were not standardized during the study period and therefore may have contributed to the delayed improvements in MI, EI, and other skin‐quality parameters. Finally, the study population consisted entirely of Fitzpatrick skin phototypes III and IV, which supports relevance to Asian skin but may limit generalizability to other skin phototypes.

Despite these limitations, the overall dataset is internally consistent and clinically meaningful. The combination of early improvements in R5 and R7, delayed improvement in R2, and later reduction in MI and EI, and favorable imaging‐based changes suggests that the device may exert a multidimensional rejuvenation effect encompassing both biomechanical and optical domains of skin aging.

## 5 Conclusion

In this prospective pilot study, a dual‐wavelength picosecond Nd:YAG laser using 1064‐nm stamping and 532‐nm toning demonstrated progressive improvement in facial skin elasticity, skin tone, and imaging‐based skin‐quality parameters in adults with facial skin aging.

These findings suggest a potential nonablative lifting effect, likely related to fractional picosecond laser‐induced dermal remodeling.

Larger controlled studies incorporating longer follow‐up, three‐dimensional facial contour assessment, and histologic or molecular correlation are warranted to define the durability, comparative efficacy, and underlying remodeling mechanisms of this treatment approach.

## Author Contributions

**Yoon Hwan Lee:** data curation, formal analysis, visualization, writing – original draft. **Sin Woo Sung:** investigation, data curation, writing – review and editing. **Ji Yeon Hong:** investigation, supervision, writing – review and editing. **Kui Young Park:** conceptualization, methodology, investigation, formal analysis, supervision, project administration, funding acquisition, writing – review and editing. All authors reviewed and approved the final version of the manuscript.

## Funding

This study was supported by Laseroptek Co. Ltd. (Seongnam, Republic of Korea). The sponsor had no role in the study design, data collection, analysis, or interpretation of the data, manuscript preparation, or the decision to submit the manuscript for publication.

## Ethics Statement

The study protocol was approved by the Institutional Review Board of Chung‐Ang University Hospital (IRB No. 2506‐016‐647) and was conducted in accordance with the ethical principles of the Declaration of Helsinki.

## Consent

Written informed consent was obtained from all participants before study enrollment, including consent for clinical photography and publication of de‐identified images.

## Conflicts of Interest

Kui Young Park serves as a consultant for Laseroptek Co. Ltd. The remaining authors declare no conflicts of interest.

## Data Availability

The datasets generated and/or analyzed during the current study are available from the corresponding author on reasonable request.
